# Hierarchically porous copper and gallium loaded sol–gel phosphate glasses for enhancement of wound closure

**DOI:** 10.1039/d5tb01945a

**Published:** 2025-11-14

**Authors:** Charlotte A. Berry, Katre Reinart, Glen J. Smales, Holly N. Wilkinson, Matthew J. Hardman, Sofia Marchesini, William Lee, Eveliny Tomás Nery, Zarrin Moghaddam, Agron Hoxha, Mónica Felipe-Sotelo, Jorge Gutierrez-Merino, Daniela Carta

**Affiliations:** a School of Chemistry and Chemical Engineering, University of Surrey Guildford GU2 7XH UK d.carta@surrey.ac.uk +44 (0)1483 689587; b Surface Technology Group, National Physical Laboratory Teddington TW11 0LW UK; c Bundesanstalt für Materialforschung und – prüfung (BAM) Berlin Germany; d Institute for Inorganic Chemistry, Graz University of Technology Stremayrgasse 9 8010 Graz Austria; e Centre for Biomedicine, Hull York Medical School, University of Hull Hull HU6 7RX UK; f Skin Research Centre, Hull York Medical School, University of York York YO10 5DD UK; g School of Biosciences and Medicine, University of Surrey Guildford GU2 7XH UK

## Abstract

In this work, we have developed hierarchically porous phosphate-based glasses (PPGs) as novel materials capable of promoting wound closure and simultaneously delivering antibacterial effects at the glass-biological tissue interface. PPGs are characterised by extended porosity, which enhances the controlled release of therapeutic ions, whilst facilitating cell infiltration and tissue growth. Two series of PPGs in the systems P_2_O_5_–CaO–Na_2_O–CuO and P_2_O_5_–CaO–Na_2_O–Ga_2_O_3_ with (CuO and Ga_2_O_3_ 0, 1, 5 and 10 mol%) were manufactured using a supramolecular sol–gel synthesis strategy. Significant wound healing promotion (up to 97%) was demonstrated using a human *ex vivo* wound model. A statistically significant reduction of the bacterial strains *Staphylococcus aureus* and *Escherichia coli* was observed in both series of PPGs, particularly those containing copper. All PPGs exhibited good cytocompatibility on keratinocytes (HaCaTs), and analysis of PPG dissolution products over a 7-day period demonstrated controlled release of phosphate anions and Ca, Na, Cu, and Ga cations. These findings indicate that Cu- and Ga-loaded PPGs are promising materials for applications in soft tissue regeneration given their antibacterial capabilities, *in vitro* biocompatibility with keratinocytes and *ex vivo* wound healing properties at the biomaterial-human tissue interface.

## Abstract 2

Hierarchically porous phosphate glass powders for wound healing. Significant wound healing promotion (up to 97%) was demonstrated using a human *ex vivo* wound model.

## Graphical abstract

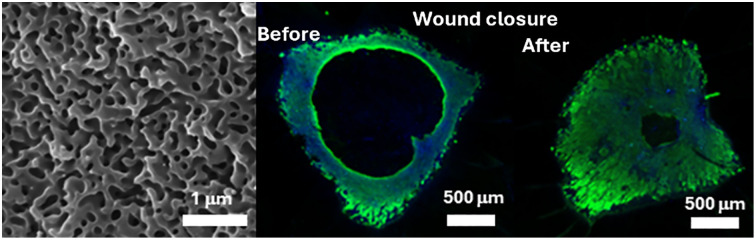

## 1. Introduction

Inorganic oxide glasses with hierarchical porosity are of great interest as biomaterials thanks to their extended porosity, high surface areas, potential for surface functionalisation, and enhancement of bioactivity.^1–3^

Depending on their sizes, pores are classified as micropores (<2 nm), mesopores (2 to 50 nm) or macropores (>50 nm).^4^ Micropores increase the surface area of the glasses, mesopores facilitate the uptake/release of therapeutic species whilst macropores promote cell attachment, movement of fluids and vascularisation; materials with this combination of porosity (hierarchical) show excellent potential for tissue regeneration.^5^

These peculiar textural properties facilitate interactions between the glasses and biological fluids, enhance their loading capability, and make them ideal materials for controlled delivery of therapeutic ions and molecules. High quantities of therapeutic species can be loaded into the pores and released in a controlled manner into the damaged site.

Extensive research has been presented on mesoporous silicate-based glasses (MSGs), predominantly as drug delivery systems and for tissue engineering applications.^6,7^ However, silicate-based glasses have relatively low solubilities which could lead to localised inflammatory responses and/or potentially contribute to long-term implant failure, limiting their use as biomaterials.^8–12^ Only recently, alternative porous inorganic oxide systems with P_2_O_5_ as a main component (porous phosphate-based glasses (PPGs)) have been presented.^12–16^

In contrast to MSGs, PPGs are fully bioresorbable, meaning they completely degrade in a physiological environment, and are eventually entirely replaced by regenerated tissues.^17^ Thanks to their complete degradation into non-toxic species, PPGs can be used simultaneously as safe degradable temporary implants and controlled delivery systems, removing the need for potentially invasive secondary surgeries.^18^ Therapeutic species released upon dissolution can have specific functions, such as antibacterial (*e.g.* Ag^+^, Cu^2+^, Zn^2+^, Ga^3+^, Ce^3+/4+^) and antioxidant activity (*e.g.* Ga^3+^, Ce^3+/4+^), promotion of wound healing and/or enhancement of cell attachment and proliferation.^19–22^ In addition, the release of therapeutic species from phosphate glasses (PGs) has been shown to modulate and direct tissue regeneration and prevent infections often occurring post-surgery.^23^ In this study, the combined antibacterial and regenerative properties of PPGs loaded with copper and gallium ions on wound healing were investigated.

PPGs cannot easily be obtained using the conventional method of melt-quenching (MQ), which involves the melting of oxide powders at high temperatures (1000–1200 °C), followed by rapid quenching. Instead, a bottom-up, in-solution method such as the sol–gel (SG) approach can be utilised in combination with supramolecular templating. MSGs prepared *via* this approach have been proven to promote material–cell interactions, exhibiting higher tissue bonding rates and cell infiltration throughout the glass scaffold.^1,24^ The SG method involves hydrolysis and polycondensation of inorganic alkoxide precursors. SG is a far more versatile technique compared to MQ, offering control over the synthesis of a broader range of compositions and the ability to form different morphologies, such as monoliths, fibres, and spheres, which can be tailored to specific applications. SG also offers even mixing of the reactants, resulting in high-purity, homogeneous glasses.^25^ Overall, SG glasses exhibit improved bioactivity compared to those prepared by MQ, owing to their inherently enhanced textural properties.^26^ The addition of surfactants to SG precursor solutions (supramolecular templating) facilitates the preparation of glasses with highly controlled porosity and enhanced surface areas.^1^ Commonly, Pluronic-type, non-ionic triblock copolymers of poly (ethylene oxide)–poly (propylene oxide)–poly (ethylene oxide) (PEO–PPO–PEO) are used for the synthesis of MSGs. At concentrations higher than the critical micellar concentration, surfactant molecules self-assemble into micelles of different shapes and sizes depending on the surfactant type, temperature and solvent used. The micelles are then removed *via* thermal treatment (calcination) or solvent exchange, generating extended porosity, with pore shapes and sizes dependent on the micelle morphologies.

Much less work has been done on PPGs compared to MSGs, given that the phosphate network is more prone to crystallisation and collapse of the porous structure upon calcination, which is required for the removal of a templating agent.^26^ Only very recently, PPGs prepared *via* SG using the surfactant Pluronic P123, in the ternary system P_2_O_5_–CaO–Na_2_O, and in the quaternary systems obtained by adding 1, 3 and 5 mol% Sr^2+^, Zn^2+^ or Cu^2+^ have been presented by Foroutan *et al*.^12–15^ In the ternary PPG system, enhanced *in vitro* properties were observed in comparison to its non-porous counterpart, namely, the kinetics of a hydroxycarbonate apatite layer deposition and the attachment and proliferation of Saos-2 osteosarcoma cells on the surface of PPGs.^12^ PPGs containing Sr^2+^ and Zn^2+^ showed effective release of these therapeutic ions upon degradation, confirming the applicability of PPGs as controlled delivery systems.^13,15^ Finally, PPGs containing copper ions exhibited antibacterial activity against both *Staphylococcus aureus* (*S. aureus*) and *Escherichia coli* (*E. coli*) with the highest efficiency observed for the highest Cu loading content (5 mol% CuO).^14^

In this work, PPGs in the systems P_2_O_5_–CaO–Na_2_O–CuO and P_2_O_5_–CaO–Na_2_O–Ga_2_O_3_ (with CuO and Ga_2_O_3_ 0, 1, 5 and 10 mol%) were prepared *via* the templated SG technique using a different templating agent, F108 instead of P123. Compared to P123, F108 has a higher hydrophilicity owing to its higher PEO : PPO ratio, which can help to improve solubility in solution. Therefore, the use of F108 could facilitate the formation of a more robust porous structure, allowing tighter control over the porosity and yielding a more homogeneous pore distribution. F108 has been utilised in this study to explore the possibility of achieving enhanced surface areas and improved porosity. Moreover, a wider range of copper loading was used to determine whether increased loading can further enhance the antibacterial activity of the PPGs.^14^

Copper is an essential element in the body, participating in several physiological and metabolic activities, including the modulation of inflammatory responses and the stimulation of the migration and proliferation of endothelial cells (vital for angiogenesis), and therefore important in the wound healing process.^27–29^

Copper-based materials have been widely used as broad-spectrum antibacterial agents, effective against both Gram-negative and Gram-positive bacteria.^19^ Potential mechanisms of action involve Cu ions engaging in nucleophilic attacks against the bacterial cell membrane or interacting with nucleic proteins/acids, thereby impairing their functions and ultimately leading to cell death.^30^ These effects are generally unspecific to bacterial type, although slightly increased potency against Gram-positive bacteria has been observed.^31^

Gallium exhibits anti-inflammatory and haemostatic properties through the activation of coagulation biological pathways, which are essential for early stages of wound healing.^32,33^

Gallium has also received interest as a broad-spectrum antibacterial agent, with its mode of action linked to iron mimicry as Ga^3+^ and Fe^3+^ share similar chemical behaviours (*e.g.* ionic radius, oxidation state).^34^ When bacteria uptake gallium in place of iron, essential iron-dependent metabolic processes can be disrupted, effectively diminishing infection rates, since iron is essential for the growth and survival of many bacterial species.^34^

Therefore, in this study, the incorporation of copper and gallium into PPGs has been specifically targeted to assess their antibacterial properties against *E. coli* and *S. aureus* (bacterial strains commonly associated with wound infections), wound healing properties through human *ex vivo* skin models and biocompatibility towards keratinocytes.

## 2. Materials and methods

### 2.1. Synthesis of PPGs

The following chemicals were used for the synthesis of the PPGs without further purification; *n*-butyl phosphate (*n*-BP, 1 : 1 molar ratio of mono- and di-butyl phosphate, [CH_3_(CH_2_)_3_O]_2_P(O)OH/[CH_3_(CH_2_)_3_O]P(O)(OH)_2_ 98%, Alfa Aesar), calcium methoxyethoxide (Ca(OCH_2_CH_2_OCH_3_)_2_ hereafter named CMOE, 20% in methoxyethanol, ABCR), sodium methoxide (NaOCH_3_, hereafter named NaOMe, 30 wt% in methanol, Aldrich), gallium(iii) nitrate hydrate (Ga(NO_3_)_3_·H_2_O, 99.9%, Aldrich), copper(ii) nitrate trihydrate (Cu(NO_3_)_2_·3H_2_O, 99%, Aldrich), ethanol (CH_3_CH_2_OH, hereafter named EtOH, 99%, Fisher), and Pluronic F108 (F108, PEO_133_PPO_50_PEO_133_*M*_n_ = 14 600 g mol^−1^, Aldrich).

**Fig. 1:**
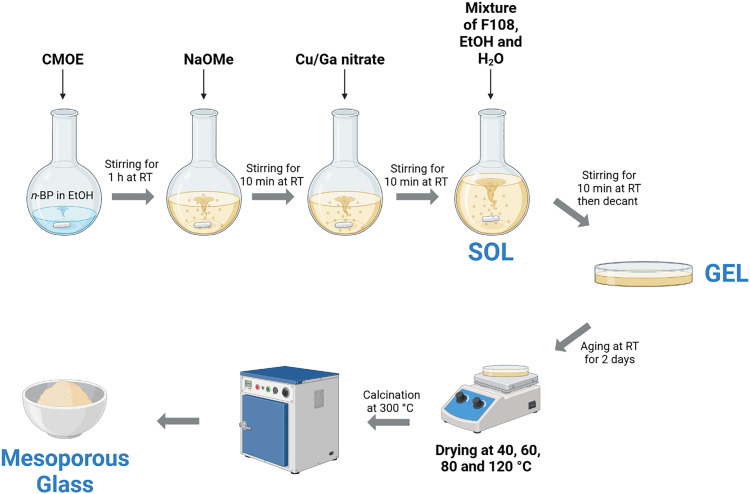
Schematic of the sol–gel synthesis of PPG-Cu*X* and PPG-Ga*X*.

A schematic of the SG preparation of the PPGs is illustrated in Fig. 1.

For the synthesis of the PPGs in the system P_2_O_5_–CaO–Na_2_O, 1.7 g of *n*-BP was added to 5 mL of EtOH in a dried round-bottomed flask and left stirring for 10 minutes (min). 3.5 g of CMOE was then added dropwise into the mixture while stirring; the solution was left stirring for 1 hour (h) before addition of 0.5 g of NaOMe; the mixture was then left under stirring for an additional 10 min. Finally, 2.5 g of F108 was dissolved in a solution of 5 mL EtOH and 2.5 mL deionised (DI) water, added to the mixture then left to stir for a further 10 min.

During the synthesis, ethanol plays a key role in achieving a miscible sol. It interacts with the alkoxide precursors primarily through alkoxy (ligand) exchange reactions, which moderate precursor reactivity, enhance chemical compatibility and stabilise the alkoxide species in solution through solvation and intermolecular interactions.^35,36^ These effects help to prevent phase separations and ensure the formation of a stable, homogeneous sol, as observed in other SG glass systems.^37,38^

For the synthesis of the quaternary PPGs, prior to the addition of the surfactant solution, the desired quantities of copper or gallium precursors were added to the mixture. For the preparation of P_2_O_5_–CaO–Na_2_O–CuO (CuO = 1, 5 and 10 mol%), 0.044 g, 0.22 g and 0.44 g of Cu(NO_3_)_2_·3H_2_O were added to the solution for the 1, 5, and 10 mol% compositions, respectively. Corresponding amounts of NaOMe were adjusted to 0.47 g, 0.34 g, and 0.17 g.

For the preparation of P_2_O_5_–CaO–Na_2_O–Ga_2_O_3_ (Ga_2_O_3_ = 1, 5 and 10 mol%), 0.0498 g, 0.249 g and 0.498 g of Ga(NO_3_)_3_·H_2_O were added for the 1, 5, and 10 mol% compositions, respectively. The NaOMe content was adjusted accordingly to 0.46 g, 0.32 g, and 0.15 g.

Finally, a solution of 3.0 g of F108 in 5.0 mL EtOH and 2.5 mL of DI water was added to the mixture containing Cu or Ga precursor.

After stirring, the solutions were transferred into a Petri dish and allowed to gel at room temperature. Gelation occurred after about 10 min and gels were left to age for 2 days (d) at room temperature (RT). The gels were further aged on a hotplate at 40 °C for 1 d, 60 °C for 3 d, 80 °C for 2 d and at 120 °C for 1 d. The PPGs were finally ground to fine powders with a pestle and mortar.

For removal of surfactant and solvents, all PPGs were calcined at 300 °C at a heating rate of 1 °C min^−1^ and a dwell time of 30 min in air. It has been reported that there is a small processing window between the loss of organics from PGs and the softening temperature at which the structure of the glass collapses.^26^ Calcination temperature was therefore chosen based on previous literature on PPGs and was performed at low heating rates to avoid pore collapse or crystallisation.^12–15^ The ternary system P_2_O_5_–CaO–Na_2_O will be hereafter named as PPG-U (unloaded) and copper and gallium loaded samples as PPG-Cu*X* or PPG-Ga*X*, respectively where X = oxide loading in mol% (1, 5 or 10).

### 2.2. Characterisation of porosity and structure of the phosphate network

X-ray powder diffraction (XRD, PANalytical X’pert diffractometer, UK) was performed on powder PPGs using Cu Kα radiation (*λ* = 1.54 Å) in transmission mode. Data collection occurred over a 2*θ* range of 10–90° with a scan step size of 0.0525°. This angular range is commonly used for SG derived PPGs and is used to confirm the amorphous nature of the phosphate network.^12,13^

Small angle X-ray scattering (SAXS) measurements were performed on the MOUSE instrument (Bundesanstalt für Materialforschung und –prüfung (BAM), Germany), where X-rays were generated from a monochromatize, microfocus X-ray source (Cu Kα, *λ* = 1.54 Å).^39^ The scattered radiation was detected using an in-vacuum, Eiger 1 M detector (Dectris, Switzerland). Powder PPGs were positioned at multiple sample-to-detector distances between 138–2507 mm, and the resulting data processed and scaled using the DAWN software package in a standardized, complete 2D correction pipeline.^40,41^ The corrected data was subsequently fitted and analysed using McSAS, a Monte Carlo method to extract form-free size distributions.^42^ Data obtained from the unloaded sample, PPG-U, are deposited on zenodo.^43^

Scanning electron microscopy (SEM) images were obtained using a TM Apreo S SEM (Thermo Fisher Scientific) at an accelerating voltage of 2–5 kV and working distance of 4 mm. The powder samples were adhered onto aluminium stubs using carbon conductive tape and graphite sputter-coated to minimise charging of the samples.

Nitrogen physisorption analysis was performed on an ASAP 2460 gas sorption instrument (Micromeritics, UK). Samples were degassed for 3 h at 200 °C in an external degas unit (FlowPrep, Micromeritics, UK) and then measured on the sorption analyser. The Brunauer–Emmett–Teller (BET) equation was used to measure the specific surface area (SSA) from nitrogen sorption isotherms.^44^ Each measurement was repeated three times, and an average BET SSA was reported. The uncertainty was calculated from the standard deviations (SDs) between individual measurements, *n* = 3.

Fourier transform infrared (FT-IR) spectroscopy was performed using a FT-IR-2000 instrument (PerkinElmer) with Spectrum 10 software. Samples were scanned at room temperature in transmission mode in the range of 3500–400 cm^−1^ with a resolution of 4 cm^−1^ and a total of 16 scans.

### 2.3. Ion release studies

10 mg of each powdered PPG was immersed in 10 mL deionised (DI) water and left in an incubator-shaker (37 °C, 220 rpm) for 4, 16, 24, 48, 72 and 168 h. For each timepoint, three separate experiments were completed to assess reproducibility (*n* = 3). DI water is a commonly used medium to investigate ion release to study the dissolution kinetics of the material.^45,46^ For analysis, the suspensions were centrifuged at 4500 rpm for 5 min, filtered to separate the powder from the solution using a 0.45 µm syringe filter (Millex, Merck Millipore) and then diluted to a 1 : 50 ratio with 2% (v/v) HNO_3_ (for trace metal analysis 68% (v/v), Fisher Scientific). Release of phosphorus, calcium, sodium, copper, and gallium ions in solution were measured as total elemental concentrations by an Agilent 4210 microwave plasma atomic emission spectrometer (MP-AES, Agilent Technologies). A multi-elemental stock solution prepared from 1000 mg L^−1^ stock solutions (Aristar, Fisher Scientific) and diluted with 2% HNO_3_ was used for calibration in the concentration range 0–100 µg mL^−1^. Emission intensities were measured at 213.6 nm (P), 396.8 nm (Ca), 588.9 nm (Na), 324.8 nm (Cu) and 287.4 nm (Ga). To correct for instrument drift during the analytical run, the emission signals for each element were normalised by ratioing a beryllium internal standard (5 µg mL^−1^, 234.9 nm, and 313.0 nm) intensity.

### 2.4. Antibacterial studies

The antibacterial properties of the PPGs at different time points were tested against *S. aureus* (NCTC 8325) and *E. coli* (K12) using the quantitative agar dilution method (ADM). Strains of *S. aureus* and *E. coli* were grown in Tryptic Soy Broth (TSB) at 37 °C in an incubator-shaker (250 rpm) until the cultures reached the mid-exponential phase of growth. The cells were then diluted to a concentration of approximately 10^6^ cells mL^−1^ in TSB containing 10 mg or 20 mg of PPG-Cu*X*/PPG-Ga*X* and placed into an incubator-shaker (37 °C, 250 rpm). After 24, 48 and 72 h of incubation, the cultures were diluted serially and spotted on Tryptic Soy Agar (TSA) in triplicates and allowed to grow at 37 °C until colonies could be observed and counted. The number of colony forming units per mL (CFUs mL^−1^) was then calculated by multiplying the number of colonies by the dilution factor and dividing this value by the amount of culture plated. Bacterial viability is expressed as log10 CFU mL^−1^ and error bars represent SDs. These antibacterial tests are commonly used in microbiology to quantify bacterial survival/growth, allowing quantitative comparisons between samples. A two-way ANOVA with Tukey's multiple comparisons test was performed for all samples at each time point to calculate statistically significant differences.

### 2.5. Cytocompatibility

The cytocompatibility of PPG-U, PPG-Cu*X* and PPG-Ga*X* on HaCaTs (spontaneously transformed keratinocytes from histologically normal skin, AddexBio, Catalog Number T0020001) was performed *via* MTT assay. HaCaTs were diluted to a concentration of approximately 10^5^ cells mL^−1^ in Dulbecco's modified Eagle medium (DMEM, Gibco) prepared with l-glutamine, streptomycin/penicillin, 2% FBS, 1 mM CaCl_2_, and seeded in 96 well tissue-treated plates. The cells, incubated at 37 °C in a 5% CO_2_ atmosphere, were allowed to grow for 24 h and then washed with PBS. Then a DMEM solution, supplemented with 0.4 mM CaCl_2_ and containing the 24 h dissolution products from the PPGs, was added to each well in a 1 : 10 ratio (10 µL in 90 µL of cell culture) in triplicates. Positive controls consisted of cells incubated with medium with no dissolution product. After 24 h incubation in the same conditions described above, MTT reagent (thiazolyl blue tetrazolium bromide, Sigma-Aldrich - 1 mg mL^−1^ in DI water) was added to each well (12 µL of MTT in 100 µL of cell culture) and the plate was incubated again for 3 h. Afterwards, the cells were washed with PBS and 100 µL of DMSO (99.9%, Sigma-Aldrich) was added to each well. The absorbance of the cultures at 570 nm was measured using a microplate reader (Fluostar-Omega, BMG LabTech, Germany). The percentage of metabolically active HaCaTs was calculated by comparing the average absorbance at 570 nm of the positive control with that of the wells where dissolution products were added. A one-way ANOVA with Dunnett's multiple comparisons test was performed to calculate statistically significant differences.

### 2.6. Human *ex vivo* wound model and whole-mount staining

Human *ex vivo* wounding and whole-mount staining were performed following the protocol presented by Wilkinson *et al*.^47^ Healthy human abdominal skin was obtained under full informed, written patient consent, institutional guidelines, and ethical approval (LREC: 17/SC/0220) from patients undergoing reconstructive surgery at Castle Hill Hospital (Hull, UK). Briefly, partial thickness wounds (2 mm) were created in the centre of skin explants (6 mm) and cultured on a nylon filter (0.45 µm) at the air : membrane interface. Standard high glucose DMEM containing 10% FBS and 1% antibiotic : antimycotic solution (Gibco) was used as a growth medium. The sterile-filtered 24 h PPG dissolution products were added to the growth media to give a 1% (v/v) final working concentration, with 10 µL of the PPG-containing media applied topically to each wound. Growth medium including 1 : 100 DI water alone (“untreated” group) was used as a control. Wound explants were then cultured at 35 °C and 5% CO_2_ for 2 d before collecting in neutral buffered formalin for whole-mount analysis.

The formalin fixed wound samples were rinsed in PBST (PBS containing 0.5% Triton X-100) prior to incubating with anti-mouse keratin 14 antibody (clone: LL002; Abcam, UK) to measure re-epithelialisation. Following primary antibody incubation, biopsies were incubated in Alexa Fluor 488-conjugated goat anti-mouse secondary antibody (Thermo Fisher Scientific) to detect keratin 14 staining in the migrating keratinocytes. The biopsies were then counterstained with 4′,6-diamidino-2-phenylindole (Thermo Fisher Scientific) and imaged on a confocal laser scanning microscope (LSM 710, Carl Zeiss, Germany) using a 2.5× objective, 405-nm diode, and 488-nm argon lasers. A one-way ANOVA with Dunnett's multiple comparisons test was performed on *ex vivo* data (comparing all groups to PPG-U), with significance determined where *P* < 0.05.

## 3. Results

### 3.1. Composition and assessment of structure *via* XRD

**Fig. 2:**
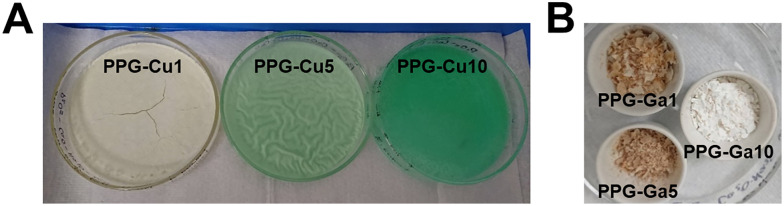
Images of (A) gels containing copper (1, 5 and 10 mol%) after 48 h of ageing at RT and (B) PPG-Ga*X* samples after calcination at 300 °C.

Representative images of the wet gels containing copper after ageing in air for 48 h, are presented in Fig. 2A, whereas images of the dried gels containing gallium after calcination in air at 300 °C are shown in Fig. 2B. Nominal compositions for PPG-Cu*X* and PPG-Ga*X* are presented in Table 1, whilst those for PPG-U are shown in Table S1.

**Table 1:** Nominal oxide compositions of PPGs in mol%

Sample	Composition (mol%)				
	P_2_O_5_	CaO	Na_2_O	CuO	Ga_2_O_3_
PPG-Cu1	48	38	13	1	—
PPG-Cu5	48	38	9	5	—
PPG-Cu10	48	38	4	10	—
PPG-Ga1	48	38	13	—	1
PPG-Ga5	48	38	9	—	5
PPG-Ga10	48	38	4	—	10

The composition of the ternary P_2_O_5_–CaO–Na_2_O system has been chosen based on previous studies on MQ and SG PGs reported in the literature, including a MQ study with a ternary diagram highlighting the glass-forming regions.^48^ Systems containing between 40–55 mol% P_2_O_5_ and 30–40 mol% CaO have demonstrated excellent bioactivity and biocompatibility.^46^ Both Ca^2+^ and Na^+^ are biologically relevant ions that play key roles in cellular function and tissue regeneration, including the wound healing process.^49^ The incorporation of CuO and Ga_2_O_3_ is expected to further enhance these properties.^50,51^

**Fig. 3:**
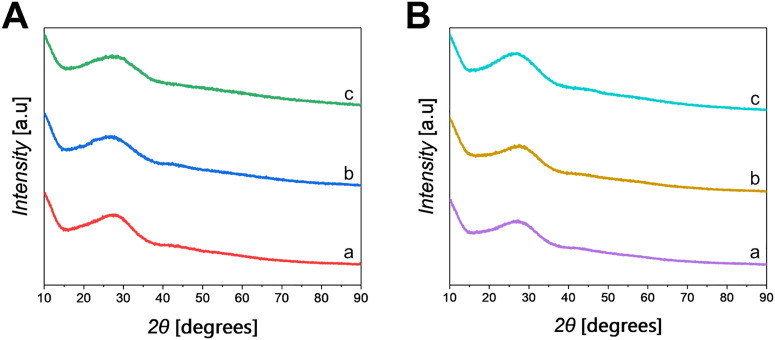
XRD patterns of A: (a) PPG-Cu1, (b) PPG-Cu5 and (c) PPG-Cu10; B: (a) PPG-Ga1, (b) PPG-Ga5 and (c) PPG-Ga10.

After calcination, all PPGs are amorphous as shown by the lack of Bragg peaks in the XRD patterns reported in Fig. 3A (PPG-Cu*X*), Fig. 3B (PPGs-Ga*X*) and Fig. S1 (PPG-U). The broad peak centred at ∼30° can be ascribed to the phosphate glass network.

### 3.2. Assessment of porosity *via* SAXS, SEM and N_2_ adsorption–desorption

During the SG synthesis, the Pluronic template (structure-directing agent) self-organises into micelles that interact with the phosphate precursor. The removal of the sacrificial Pluronic template *via* calcination at 300 °C resulted in hierarchically porous PGs. The porous structure was investigated using three complementary techniques: SAXS, SEM, and N_2_ physisorption at 77 K, allowing investigation of all ranges of pore sizes.

**Fig. 4:**
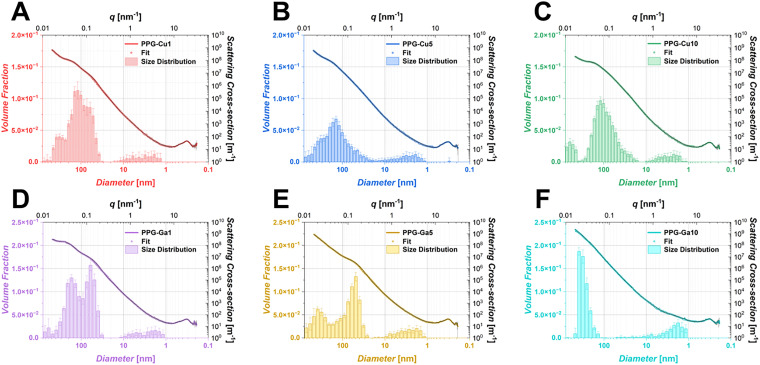
Fitted SAXS data for (A) PPG-Cu1, (B) PPG-Cu5, (C) PPG-Cu10; (D) PPG-Ga1, (E) PPG-Ga5 and (F) PPG-Ga10.

SAXS fits and corresponding histograms for PPG-Cu*X* and PPG-Ga*X* samples showing structural size distributions are presented in Fig. 4. PPGs present hierarchical porosity with a simultaneous presence of micro-, meso- and macropores, with the mean pore diameter for distinct populations (primary, secondary and tertiary) presented in Table 2. Data for PPG-U is presented in Fig. S2 and Table S2.

**Table 2:** Mean diameter of pore populations from PPG-Cu*X* and PPG-Ga*X* derived from fits of SAXS data using Monte-Carlo methods

Sample	Size distribution range (nm)		
	Population 1 (primary)	Population 2 (secondary)	Population 3 (tertiary)
PPG-Cu1	102 ± 1	4 ± 1	286 ± 4
PPG-Cu5	104 ± 1	5 ± 1	278 ± 4
PPG-Cu10	98 ± 1	4 ± 1	467 ± 4
PPG-Ga1	63 ± 1	4 ± 1	183 ± 4
PPG-Ga5	73 ± 1	4 ± 1	371 ± 6
PPG-Ga10	—	3 ± 1	335 ± 5

In the analysis of PPG-U, a primary population of structures was identified between 20 nm and 200 nm, with a mean diameter of 60 nm. This primary population is accompanied by a secondary population of smaller structures (micro-/mesopores), ranging from 2 nm to 12 nm (mean diameter ∼4 nm), occupying a significantly lower overall volume fraction. These observed features are likely associated with the micro-/mesoporous nature of the PPG structure.

Upon loading with Cu, the secondary population remains largely unchanged in both size and volume fraction across all loading levels, with an average pore diameter of ∼4 nm. In contrast, the primary population undergoes notable changes. For PPG-Cu1, the volume fraction of the primary population decreases and becomes more polydisperse. This primary population exhibits a, somewhat, overlapping bimodal distribution, with one mode around 60 nm (similar to PPG-U) and another around 120 nm, resulting in an overall mean diameter of 102 nm. A tertiary population, with a mean diameter of 286 nm, also emerges in this sample. Similar trends are observed for samples with higher Cu content (PPG-Cu5 and PPG-Cu10), where increased loading shifts the primary pore size from the original 60 nm to an 104 and 98 nm, respectively. This shift suggests a transition towards larger pore sizes at the expense of the smaller primary pores, leading to a reduction in bimodality as Cu content increases. All loaded samples also display larger tertiary structures compared to PPG-U.

For Ga-loaded samples, the secondary population decreases in size compared to PPG-U, with an average diameter reduced to approximately 4 nm across all Ga loading contents. The primary population also undergoes significant changes. In PPG-Ga1, a distinct bimodal distribution is observed, with mean pore diameters of 63 nm and 183 nm. As Ga loading increases (PPG-Ga5), the bimodal nature becomes even more prominent, featuring average diameters of 73 nm and 371 nm. However, for PPG-Ga10, the smaller pore population around 60 nm is almost completely lost, leaving only larger pores with an average diameter of 335 nm.

**Fig. 5:**
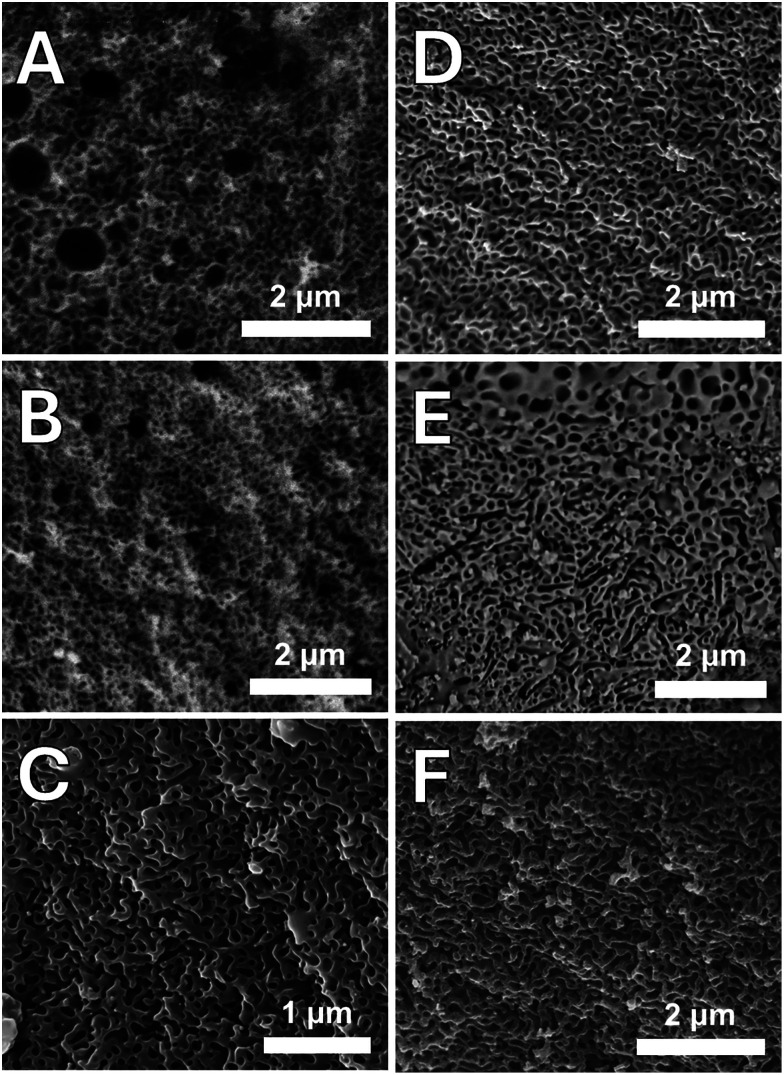
SEM images of (A) PPG-Cu1, (B) PPG-Cu5, (C) PPG-Cu10, (D) PPG-Ga1, (E) PPG-Ga5 and (F) PPG-Ga10.

Presence of porosity is confirmed by the SEM images of all PPG-Cu*X* and PPG-Ga*X* samples, reported in Fig. 5. SEM shows extended macroporosity across all PPGs (pore diameters in the range 100–450 nm) and some evidence of mesoporosity (pore diameter 40–50 nm). Interestingly, the presence of microspheres was encountered with PPG-U (Fig. S3A, sphere diameters 1–3 µm) and PPG-Ga5 (Fig. S3B, sphere diameters 1–4 µm).

**Fig. 6:**
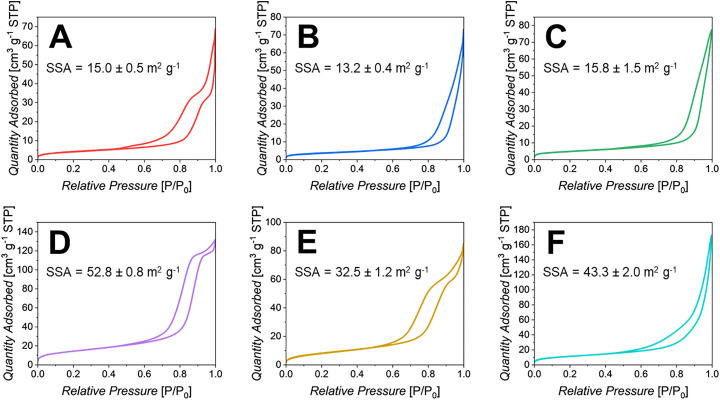
N_2_ sorption isotherms of (A) PPG-Cu1, (B) PPG-Cu5, (C) PPG-Cu10, (D) PPG-Ga1, (E) PPG-Ga5 and (F) PPG-Ga10. SSAs of each sample (m^2^ g^−1^) determined by the BET model, with SDs (*n* = 3).

Given that both SAXS and SEM show extended porosity, N_2_ adsorption–desorption analysis at 77 K was performed to provide quantitative information on SSAs of all PPGs. The N_2_ sorption isotherms and SSAs calculated *via* the BET model for PPG-Cu*X* and PPG-Ga*X* are shown in Fig. 6. For PPG-U, this information can be found in Fig. S4.

Isotherms for all PPGs can be classified as type IV, indicative of mesoporous materials due to the occurrence of capillary condensation, which leads to the formation of a hysteresis loop. The shape of the hysteresis loop can be used to further describe pore geometries.^4^

All hysteresis loops appear to be a mixture of type H1 and type H2, indicating a heterogeneous porous network. Type H1 is predominant for PPG-U, PPG-Cu5, PPG-Cu10 and PPG-Ga10, suggesting more of a cylindrical mesoporous structure, as observed in other PPG systems using P123 as a template.^15^ Type H2, characterised by a ‘stepped’ hysteresis loop, is more evident in PPG-Cu1, PPG-Ga1 and PPG-Ga5. This type of loop is indicative of inaccessible pores (potential cavitation effects or ink-bottle porosity), and suggests more of a complex, disordered network with interconnected porosity of varying sizes.

A decrease in SSA was observed upon loading with Cu or Ga, with the highest SSA observed for PPG-U (64.1 m^2^ g^−1^). In particular, all PPG-Cu*X* exhibited relatively low surface areas ranging from 13.5 to 15.8 m^2^ g^−1^, lower than all PPG-Ga*X* whose SSA varies from 32.3 to 52.8 m^2^ g^−1^.

### 3.3. FT-IR spectroscopy

**Fig. 7:**
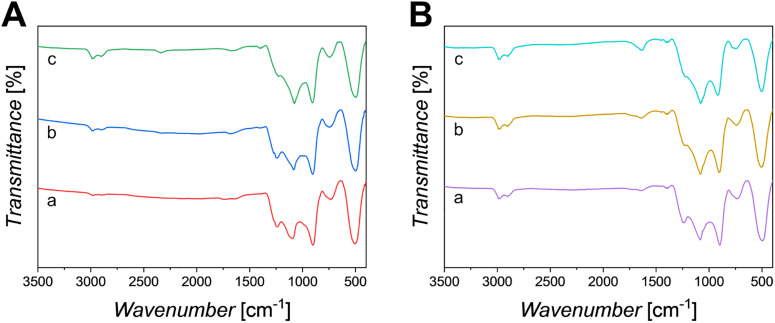
FT-IR spectra of (A): (a) PPG-Cu1, (b) PPG-Cu5 and (c) PPG-Cu10; (B): (a) PPG-Ga1, (b) PPG-Ga5 and (c) PPG-Ga10.

FT-IR spectra of PPG-Cu*X* and PPG-Ga*X* are reported in Fig. 7A and B, respectively, with PPG-U presented in Fig. S5 for comparison. All bands have been assigned according to previous studies on SG PPGs.^13–15^ All spectra show similar bands and are characteristic of an amorphous phosphate glass network, with no observed shifts between all PPGs.

Q^*n*^ notation can be used to describe the nature of the P–O vibrations in the phosphate units, where *n* represents the number of bridging oxygens (*n* = 0, 1, 2 or 3). Bands at 500 cm^−1^, 750 cm^−1^ and 900 cm^−1^ are assigned to deformation, symmetric and asymmetric stretching of the Q^2^ (P–O–P) units, respectively. The bands at around 1100 cm^−1^ and 1250 cm^−1^ are assigned to asymmetric stretching of terminal Q^1^ units *υ*_as_ (PO_3_)^2−^ and to non-bridging out-of-chain Q^2^ units *υ*_as_ (PO_2_)^−^, respectively. Weak bands arising from the stretching of C–H groups of residual organic residues remaining after calcination, *υ*(C–H), can be observed ∼2900 cm^−1^.

The band at 1100 cm^−1^ appears to be more pronounced as the loading content of either Cu or Ga increases, with a concomitant decrease in the bands at 900 cm^−1^ and 1250 cm^−1^.

### 3.4. Ion release studies

**Fig. 8:**
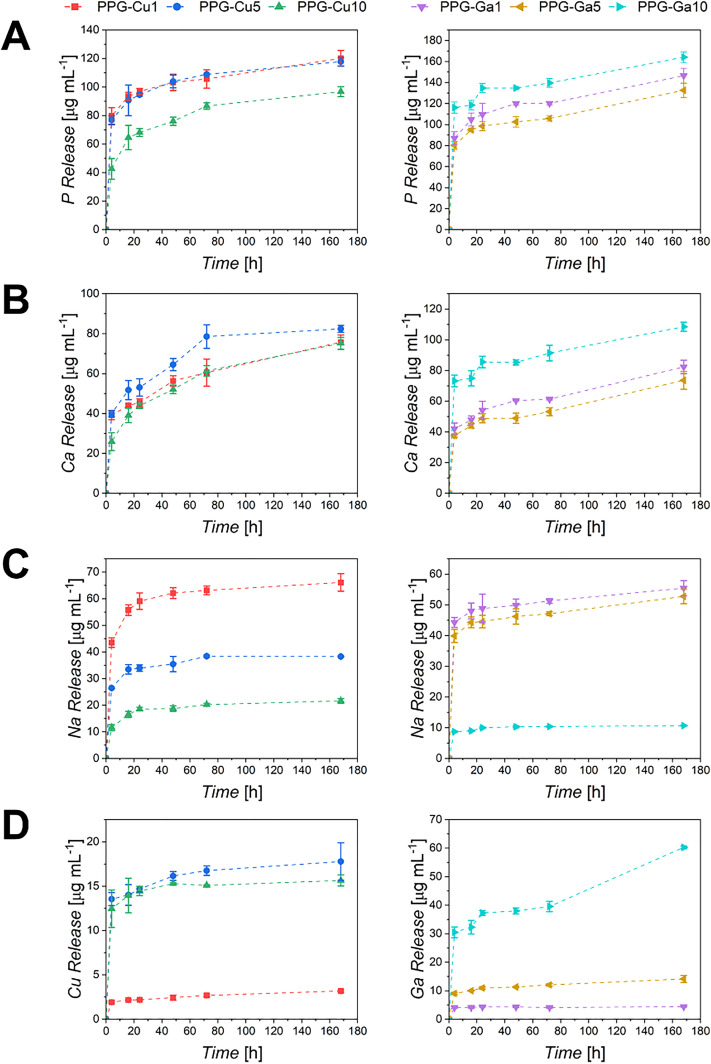
Release of (A) phosphorus, (B) calcium, (C) sodium and (D) copper/gallium from PPG-Cu*X* (left) and PPG-Ga*X* (right) following immersion in DI water for up to 7 d. Error bars correspond to ± SD (*n* = 3). Each sample has a specific symbol as defined in the legend; dashed lines are a guide for the eye.

Dissolution of all PPG-Cu*X* and PPG-Ga*X* in DI water was investigated for up to 7 d. The release profiles of phosphate anions and calcium, sodium, copper and gallium cations, quantified utilising MP-AES, are reported in Fig. 8.

All species show a high rate of release within the first 3 h, followed by a much slower, sustained release over the following time points. Release profiles of P and Ca for PPG-Cu*X* and PPG-Ga*X* respectively follow similar trends over 7 d. For both species released from PPG-Cu*X*, PPG-Cu1 and PPG-Cu5 exhibit the highest release of both P and Ca (P = ∼120 µg mL^−1^ and Ca = ∼85 µg mL^−1^), whilst PPG-Cu10 exhibits the lowest (P = ∼95 µg mL^−1^ and Ca = ∼75 µg mL^−1^) after 7 d. Contrastingly, for the PPG-Ga*X* series there is a much more distinguishable difference between the samples, with the release of P and Ca the highest for PPG-Ga10 (P = ∼165 µg mL^−1^ and Ca = ∼110 µg mL^−1^), and the lowest for PPG-Ga5 after 7 d (P = ∼130 µg mL^−1^ and Ca = ∼70 µg mL^−1^).

For both PPG series a decrease in Na release can be observed with an increase in either Cu or Ga loading. This can be explained considering that Na was substituted with either Cu or Ga during the synthesis. Therefore, as the Cu or Ga loading increases, there is a decrease in the Na content available to be released.

As expected, the release of Cu or Ga ions increases with the amount initially loaded into the sample, as there is a higher concentration of these elements available to be released. Similar trends have been reported previously in Sr and Zn-loaded PPGs.^13,15^

PPG-Ga*X* display a more defined, stepped increase in Ga release, directly correlated to the nominal composition. In contrast, the Cu release of PPG-Cu5 is very similar to that of PPG-Cu10 when accounting for errors which was unexpected given the clear decrease in Na release in these samples.

Although the nominal compositions of PPG-Cu10 and PPG-Ga10 are identical, the release of Cu ions is much lower than that of Ga ions over 7 d and the release of PPG-Ga5 is much more similar to the release of PPG-Cu5/PPG-Cu10 than that of PPG-Ga10. As mentioned previously, PPG-Cu5 and PPG-Cu10 have similar release, after 24 h, ∼14 µg mL^−1^ Cu is released from both samples, with the highest Cu release observed after 7 d, reaching only ∼17 µg mL^−1^ for PPG-Cu5. In comparison, PPG-Ga10 has a much greater, sustained release, with PPG-Ga10 releasing ∼37 µg mL^−1^ Ga after 24 h, and after 7 d, ∼60 µg mL^−1^.

### 3.5. Antibacterial assays

**Fig. 9:**
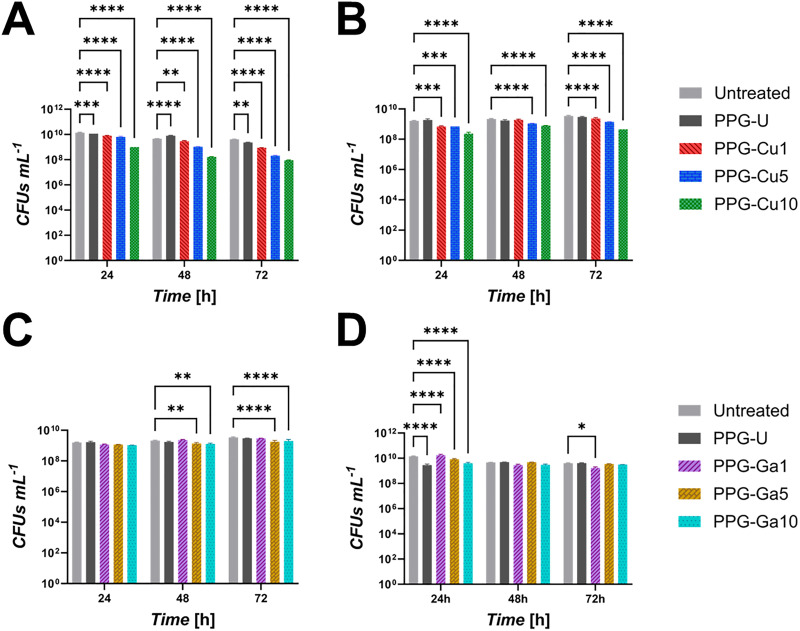
Antibacterial activity of PPG-Cu*X* (A and B) and PPG-Ga*X* (C and D) against *S. aureus* (A and C) and *E. coli* (B and D). Viability of the strains is measured by colony forming units (CFU) counts 24, 48 and 72 h after inoculation with dissolution products from each PPG. Error bars represent the SD over triplicates (*p < 0.05; **p < 0.01; ***p < 0.001; ****p < 0.0001).

The antibacterial effects of dissolution products from PPG-Cu*X* on *S. aureus and E. coli* are shown in Fig. 9A and B, respectively, with the effects of dissolution products from PPG-Ga*X* on *S. aureus and E. coli* shown in Fig. 9C and D, respectively.

Results show that after just 24 h of incubation, PPG-Cu10 has the strongest effect against *S. aureus*, causing a ten-fold reduction in viable cell count, from 10^10^ to around 10^9^ CFUs (Fig. 9A). After 24 h of incubation, a decline on the bacterial density can be observed for all PPG-Cu*X*. After 72 h, a reduction in the bacterial density is observed, correlating with increased Cu content.

PPG-Cu5 and PPG-Cu10 also show significant activity against *E. coli* after 24 h (Fig. 9B) in similar proportions to that observed against the previous strain, which is maintained up to 72 h. The culture incubated with PPG-Cu1 shows significant decline after 72 h.

Regarding PPG-Ga*X*, a decline of *S. aureus* cultures is observed after 48 h of incubation in the presence of PPG-Ga5 and PPG-Ga10; the decline is also maintained and becomes more significant after 72 h (Fig. 9C). The antibacterial activity is inconclusive when challenged against *E. Coli*. (Fig. 9D).

### 3.6. Cytocompatibility (HaCaTs)

**Fig. 10:**
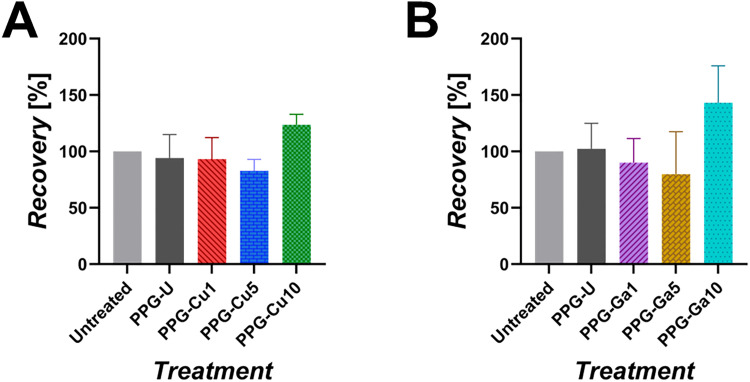
MTT assay analysis after 24 h incubation of HaCaTs exposed to 24 h dissolution products of (A) PPG-Cu*X* and (B) PPG-Ga*X*. Control = untreated cell medium. Error bars represent the SD over triplicates.^14,52,53^

Cytocompatibility of the dissolution products of PPG-U, PPG-Cu*X* and PPG-Ga*X* on HaCaTs were investigated using the MTT assay. No statistically significant effects on the viability of HaCaTs was observed for any of the dissolution products resulting from PPG-U, PPG-Cu*X* (Fig. 10A) and PPG-Ga*X* samples (Fig. 10B).

However, it is interesting to notice that in both series, the increase in recovery % is particularly high for PPG-Cu10 (123%) and PPG-Ga10 (143%) samples. PPG-U values are similar (94%) and (102%) in the two series. PPGs loaded with Cu and Ga lower than 10 mol% have similar values within the two series, regardless of the type of metal (Cu or Ga), more specifically, PPG-Cu1 (93%), PPG-Cu5 (83%) PPG-Ga1 (90%) and PPG-Ga5 (80%).

### 3.7. Human *ex vivo* wound model and whole-mount staining

**Fig. 11:**
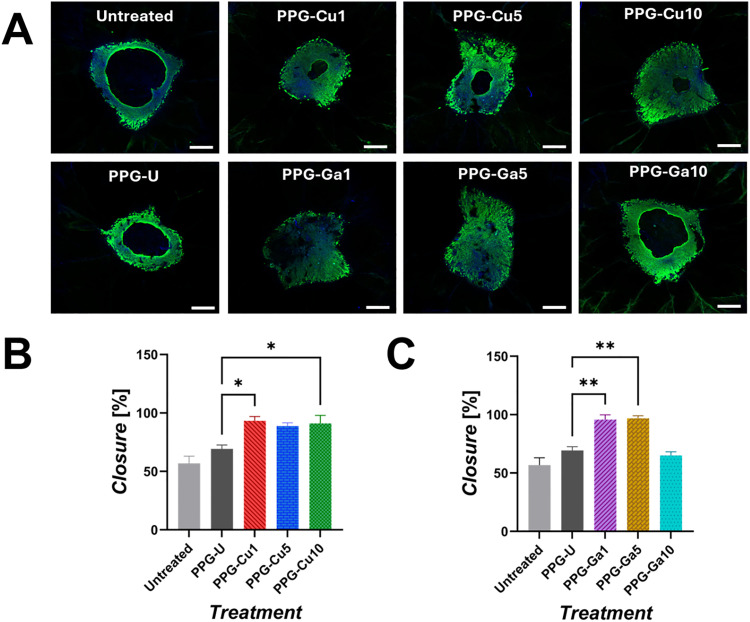
(A) Representative confocal images of whole-mount stained *ex vivo* human skin wounds following 48 h treatment with PPG dissolution products. DAPI = nuclei. Alexa Fluor 488 = keratin 14. Scale bar = 500 µm. (B) Average % wound closure of healthy human skin following treatment with PPG-Cu*X* dissolution products. (C) Average % wound closure of healthy human skin following treatment with PPG-Ga*X* dissolution products. Data shown is mean + SD (*n* = 3 per group). One-way ANOVA followed by Dunnett's *post-hoc* test was used comparing each sample to PPG-U where * = *p* < 0.05 and ** = *p* < 0.01. The “untreated” group depicts growth media with 1 : 100 DI water as a vehicle control.

A translationally relevant human *ex vivo* skin wound model was used to assess the wound healing promoting effects of PPG dissolution products over a period of 48 h (Fig. 11). The *ex vivo* skin wounds were treated with dissolution products from PPG-Cu*X* and PPG-Ga*X* and healing was assessed *via* keratin 14 whole-mount staining. Representative images from each treatment group are shown in Fig. 11A. The 24 h dissolution products of PPG-Cu*X* increased wound healing rates *vs*. that of PPG-U (Fig. 11B). These results were significant for PPG-Cu1 and PPG-Cu10 which showed a 24% and 21% increase in healing *vs*. PPG-U, respectively. Similarly, the 24 h dissolution products of PPG-Ga*X* also accelerated wound closure in the *ex vivo* human skin *vs*. PPG-U (Fig. 11C), which was significant for both PPG-Ga1 (96% *vs*. 69%) and PPG-Ga5 (97% *vs*. 69%). However, a dose-dependent effect was observed, where PPG-Ga10 no longer accelerated healing in the model, indicating that the maximum optimum concentration for Ga loading is around 5 mol%.

## 4. Discussion

SEM images show extended macroporosity across all PPGs (pore diameters in the range 100–450 nm) and some evidence of mesoporosity. However, imaging mesopores on the lower end of the range (∼2–10 nm) using SEM proves largely difficult given that the high energy electron beam tends to cause damage to the surface of the PPGs, therefore causing collapse of the mesoporous structure.^15^ This also occurs with the alternative technique of transmission electron microscopy which is commonly used for imaging on this scale.

Therefore, SAXS, a technique that allows probing of pore sizes ranging from approximately 1 to 600 nm, was used to complement SEM imaging. SAXS is the ideal technique for the analysis of hierarchically porous materials, offering advantages over other techniques used for the assessment of porous structures such as sorption measurements, as it probes electron density contrast within a material, instead of relying on accessible and permeable porous networks.^54^

SAXS has shown that PPG-U has a main population of pores ∼60 nm and a smaller one ∼4 nm. PPG-Cu*X* maintains the smallest population ∼4 nm; PPG-Cu1 is mainly bimodal (∼60 and 120 nm) with a smaller population of bigger pores at ∼286 nm. In PPG-Cu5/10 the bimodality is reduced with a shift towards larger pores being observed (larger population ∼300 nm). Similarly to PPG-Cu1, PPG-Ga1 is mainly bimodal (∼63 and 183 nm), but in PPG-Ga5 the bimodality becomes more prominent, with PPG-Ga10 only showing larger pores. These findings suggest that loading with Cu or Ga induces a shift in the primary pore population towards larger sizes, resulting in more polydisperse and overlapping pore geometries. The increased polydispersity and changes in volume fraction indicate a less ordered pore system in the loaded samples, in comparison to PPG-U.

To further analyse the morphology of PPGs, sorption analysis was performed to complement SEM imaging and SAXS analysis. However, it must be noted that SAXS analysis allows assessment of the overall porous structure whilst sorption analysis can only reflect the portion of porosity that the N_2_ can access, which could only be a fraction of the total porosity.

The fact that BET SSAs of PPG-Cu*X* and PPG-Ga*X* are quite low, suggests that not all pores are fully accessible to the N_2_ probing gas; even though the pores have been identified *via* SAXS, the fact that they are inaccessible limits adsorption and makes desorption more difficult.

PPG-U shows a higher SSA compared to other PPGs. This could be attributed to the higher volume fraction of the primary pore population averaged ∼60 nm, compared to the other samples observed *via* SAXS. It has to be noted that the surface area of PPG-U (∼64 m^2^ g^−1^) is lower than the one observed for PPG-U synthesised using a different surfactant, P123 (∼124 m^2^ g^−1^).^12^ This is largely attributed to the differences in the drying process between the two studies, however the surfactant type could also contribute to this.

Another interesting observation is that PPG-Cu*X* exhibited SSAs lower than all PPG-Ga*X*. This difference could be explained based on SAXS results, which revealed smaller pores in PPG-Ga*X* (∼3.5 nm and ∼65 nm) compared to PPG-Cu*X* (∼4.5 nm and ∼100 nm), this would therefore contribute more considerably to the SSA. This observation could also explain the difference in the release of Cu or Ga ions between PPG-Cu*X* and PPG-Ga*X*. Despite the fact that Cu and Ga ion release increases with their loading in both cases, the increase is noticeably more pronounced in PPG-Ga*X* than in PPG-Cu*X*, which can likely be attributed to the increased SSAs observed in PPG-Ga*X*, leading to a higher extent of dissolution.

Besides morphological analysis, a qualitative structural analysis was performed *via* FT-IR. The asymmetric stretching of terminal Q^1^ units *υ*_as_ (PO_3_)^2^ appears to be more pronounced as the loading content of either Cu or Ga increases, with a concomitant decrease in the Q^2^ units. This suggests that loading of the system with Cu/Ga could be causing disruption in the phosphate chains, leading to a lower degree of overall network connectivity. FT-IR analysis correlates with previous studies by Foroutan *et al.*, who employed both FT-IR and ^31^P solid state nuclear magnetic resonance to characterise loaded SG PGs.^13–15^ These studies reported mainly Q^1^ units with a smaller proportion of Q^2^ species, consistent with the present findings, and supporting the use of FT-IR to assess Q-species in such systems. As the structure of PPGs is related to ion release, identification of species released in DI water was performed *via* MP-AES. Ion release data could help to explain the antibacterial activity of the PPGs. In our study, PPG-Cu5 and PPG-Cu10 have similar release after 24 h, ∼14 µg mL^−1^. In a previous study, dissolution products released from a series of Cu-loaded PGs prepared *via* an aqueous method proposed by Foroutan *et al.* containing between 18–37 µg mL^−1^ Cu^2+^ demonstrated a rapid bactericidal effect when tested against *S. aureus* with increased antimicrobial behaviours with increased Cu loading.^51^ Similarly, ionic products from Cu-loaded meso/macroporous bioactive scaffolds prepared *via* Wu *et al.* (14.2–152.7 µg mL^−1^ Cu) highlighted enhanced angiogenesis potential, antimicrobial properties and promotion of osteogenic differentiation through the stimulation of bone-related gene expression in comparison to unloaded scaffold ionic products.^55^

PPG-Ga*X* have a much greater release, with PPG-Ga10 releasing ∼37 µg mL^−1^ Ga after 24 h. Valappil *et al.* presented MQ-derived PGs containing up to 5 mol% Ga_2_O_3_, and their dissolution in DI water over 120 h.^50^ Sustained Ga release was observed with gallium concentrations up to 39 µg mL^−1^ being recorded for the sample containing 5 mol% Ga^3+^ after 120 h. Disk diffusion assays were carried out to assess bactericidal effects of PGs against Gram-negative (*E. coli* and *P. aeruginosa*) and Gram-positive (*S. aureus*, *C. difficile)* bacteria. All samples exhibited antimicrobial properties for both Gram-negative and Gram-positive bacteria, with small effects on *C. difficile* and methicillin-resistant *S. aureus* attributed to the release of Ga^3+^.^50^

These results suggest that both PPG-Cu*X* and PPG-Ga*X* possess some antibacterial potential which can be enhanced with increasing loading of the specific antibacterial ion (copper or gallium). It also suggests that the strength of this property is dependent on the characteristics of the cell wall of the bacteria being challenged, that is, if the bacterium is Gram-positive (*S. aureus*) or Gram-negative (*E. coli*), or it can result from other differences, intrinsic of each species being challenged.^56–58^

Whilst the concentration in solution of copper and gallium ions are mainly affecting antibacterial properties, the type and concentration of all ions released upon dissolution (Ca^2+^, Na^+^ and phosphate anions) could affect cytocompatibility results. In order to fully explain the particularly high recovery % observed for PPG-Cu10 and PPG-Ga10, further investigation would be required to assess the effect on HaCaTs of other ions present in the dissolution products.

Overall, cytocompatibility data demonstrates that PPG-Cu*X* and PPG-Ga*X* present good cytocompatibility towards keratinocytes, given that the recovery % for all samples is higher than 70%. This also suggests that any residual material that might remain after the calcination stage (C or nitrates), has no detrimental effect on cell viability.

Given the excellent cytocompatibility towards HaCaTs, *ex vivo* testing on human skin was performed to investigate the wound healing capability of PPGs.

Results show that all samples exhibit excellent potential to be utilised for wound healing applications, with up to 97% wound closure observed following treatment with PPG-Ga5 dissolution products and nearly all treatments showing significant improved closure compared to the PPG-U treatment.

Within the Cu-loaded PPG series, significant increases in wound closure were observed for PPG-Cu1 and PPG-Cu10 treatments only, while PPG-Cu5 also visually promoted closure, despite not reaching a statistical significance. Interestingly, PPG-Cu5 and PPG-Cu10 contain similar copper contents, as quantified using MP-AES (∼14 µg mL^−1^) suggesting that the observed differences in closure cannot solely be attributed to Cu^2+^. Instead, a synergistic contribution from the other released ions in the solution is likely. Between the two samples, Ca^2+^ concentration is comparable (∼48 µg mL^−1^) whereas phosphates and Na^+^ concentrations differ more substantially (95 *vs*. 68 µg mL^−1^ P and 34 *vs*. 19 µg mL^−1^ Na^+^ respectively). The release of network ions such as phosphates, Ca^2+^ and Na^+^ may also enhance wound closure, as these species are broadly acknowledged to support cell metabolism and tissue repair.^17,59^

In the Ga-loaded series, wound closure was more pronounced with treatments of PPG-Ga1 and PPG-Ga5 dissolution products, whereas treatment with PPG-Ga10 had little effect. This again corresponds well with the MP-AES data, PPG-Ga1 and PPG-Ga5 have similar levels of phosphate (∼110 and 99 µg mL^−1^), Ca^2+^ (∼54 and 48 µg mL^−1^), Na^+^ (∼48 and 45 µg mL^−1^) and Ga^3+^ (∼4 and 11 µg mL^−1^), suggesting that these compositions provide a good balance for promoting wound closure in the *ex vivo* skin model. In contrast, PPG-Ga10 releases higher concentrations of phosphates, Ca^2+^ and Ga^3+^ (134, 86 and 37 µg mL^−1^ respectively) and a lower concentration of Na^+^ (∼10 µg mL^−1^). This could therefore be used to explain the lack of wound closure observed when using PPG-Ga10 as a wound treatment. The detrimental effect on wound closure is more likely caused by the elevated Ga^3+^ concentration rather than the small decrease in Na^+^, consistent with dose-dependent cytotoxicity reported in the literature.^60^ For example, a PG containing 7 mol% Ga_2_O_3_ (releasing ∼25 µg mL^−1^ Ga^3+^ into solution) exhibited only 34% cell viability against bone marrow stromal cells, attributed to the high gallium concentration.^61^

Overall, testing of PPG-Cu*X* and PPG-Ga*X* dissolution products on an *ex vivo* human skin model demonstrates their excellent potential for wound closure applications.

## 5. Conclusions

A series of hierarchically porous phosphate-based glasses in the P_2_O_5_–CaO–Na_2_O–MO system (M = Cu or Ga) were successfully prepared *via* the templated SG method using the templating agent, Pluronic F108. All samples were confirmed to be amorphous, with expected structure and extended porosity of different shapes and sizes, observed *via* SEM. SAXS analysis has shown that PPG have a micro-/mesoporous nature and that loading PPGs with Cu or Ga result in larger pore sizes, and a more polydisperse porosity. This result is confirmed by SEM analysis which shows extended macroporosity across all PPGs (pore diameters in the range 100–450 nm) and evidence of mesoporosity (pore diameter 40–50 nm).

Dissolution studies demonstrated a sustained release of phosphorus, calcium, sodium, copper, and gallium ions over 7 d. Dissolution products from all glasses showed no toxicity against keratinocytes; in addition, dissolution products from both PPG-Cu*X* and PPG-Ga*X* showed antibacterial activity *vs*. both *E. coli* and *S. aureus*.

When applied to a translationally relevant living human skin *ex vivo* wound model, dissolution products from all PPGs demonstrated significant wound healing (up to 97% closure) promoting effects compared with the control sample (69% closure).

The novel materials presented in this work opens new horizons in designing the next-generation of non-siliceous materials, with great potential in simultaneous wound promotion, antibacterial activity and controlled delivery systems.

## Conflicts of interest

There are no conflicts to declare.

## Supplementary Material

TB-013-D5TB01945A-s001

## Data Availability

The data supporting this article have been included as part of the supplementary information (SI). Supplementary information: additional information on PPG-U: XRD pattern, nominal composition, fitted SAXS data; mean diameters of pore populations, N_2_ sorption isotherm and FT-IR spectra. SEM image of PPG-U and PPG-Ga5 microspheres. See DOI: https://doi.org/10.1039/d5tb01945a.

## References

[cit1] Izquierdo-Barba I., Vallet-Regí M. (2015). Mesoporous Bioactive Glasses: Relevance of Their Porous Structure Compared to That of Classical Bioglasses. Biomed. Glasses.

[cit2] Moritz M., Geszke-Moritz M. (2015). Mesoporous Materials as Multifunctional Tools in Biosciences: Principles and Applications. Mater. Sci. Eng. C.

[cit3] Yan X., Yu C., Zhou X., Tang J., Zhao D. (2004). Highly Ordered Mesoporous Bioactive Glasses with Superior In Vitro Bone-Forming Bioactivities. Angew. Chem., Int. Ed..

[cit4] Thommes M., Kaneko K., Neimark A. V., Olivier J. P., Rodriguez-Reinoso F., Rouquerol J., Sing K. S. W. (2015). Physisorption of Gases, with Special Reference to the Evaluation of Surface Area and Pore Size Distribution (IUPAC Technical Report. Pure Appl. Chem..

[cit5] Baino F., Fiorilli S., Vitale-Brovarone C. (2016). Bioactive Glass-Based Materials with Hierarchical Porosity for Medical Applications: Review of Recent Advances. Acta Biomater..

[cit6] Vallet-Regí M., Balas F., Arcos D. (2007). Mesoporous Materials for Drug Delivery. Angew. Chem., Int. Ed..

[cit7] López-Noriega A., Arcos D., Izquierdo-Barba I., Sakamoto Y., Terasaki O., Vallet-Regí M. (2006). Ordered Mesoporous Bioactive Glasses for Bone Tissue Regeneration. Chem. Mater..

[cit8] Xia W., Chang J. (2006). Well-Ordered Mesoporous Bioactive Glasses (MBG): A Promising Bioactive Drug Delivery System. J. Controlled Release.

[cit9] Zhang X., Zeng D., Li N., Wen J., Jiang X., Liu C., Li Y. (2016). Functionalized Mesoporous Bioactive Glass Scaffolds for Enhanced Bone Tissue Regeneration. Sci. Rep..

[cit10] Matic T., Daou F., Cochis A., Barac N., Ugrinovic V., Rimondini L., Veljovic D. (2024). Multifunctional Sr,Mg-Doped Mesoporous Bioactive Glass Nanoparticles for Simultaneous Bone Regeneration and Drug Delivery. Int. J. Mol. Sci..

[cit11] Wang X., Li W. (2016). Biodegradable Mesoporous Bioactive Glass Nanospheres for Drug Delivery and Bone Tissue Regeneration. Nanotechnology.

[cit12] Foroutan F., Kyffin B. A., Abrahams I., Corrias A., Gupta P., Velliou E., Knowles J. C., Carta D. (2020). Mesoporous Phosphate-Based Glasses Prepared via Sol–Gel. ACS Biomater. Sci. Eng..

[cit13] Foroutan F., Kyffin B. A., Abrahams I., Knowles J. C., Sogne E., Falqui A., Carta D. (2020). Mesoporous Strontium-Doped Phosphate-Based Sol-Gel Glasses for Biomedical Applications. Front. Chem..

[cit14] Foroutan F., Kyffin B. A., Nikolaou A., Merino-Gutierrez J., Abrahams I., Kanwal N., Knowles J. C., Smith A. J., Smales G. J., Carta D. (2023). Highly Porous Phosphate-Based Glasses for Controlled Delivery of Antibacterial Cu Ions Prepared via Sol–Gel Chemistry. RSC Adv..

[cit15] Foroutan F., Abrahams I., Smales G. J., Kanwal N., Di Pasquale R., Knowles J. C., Andy S., Carta D. (2024). A Sol-Gel Templating Route for the Synthesis of Hierarchical Porous Calcium Phosphate
Glasses Containing Zinc. Ceram. Int..

[cit16] Li C., Wang C., Boccaccini A. R., Zheng K. (2023). Sol-Gel Processing and Characterization of Binary P2O5-CaO and Ternary P2O5-CaO-Li2O Mesoporous Phosphate Bioactive Glasses. J. Non-Cryst. Solids: X.

[cit17] Knowles J. C. (2003). Phosphate Based Glasses for Biomedical Applications. J. Mater. Chem..

[cit18] Lakhkar N. J., Lee I.-H., Kim H.-W., Salih V., Wall I. B., Knowles J. C. (2013). Bone Formation Controlled by Biologically Relevant Inorganic Ions: Role and Controlled Delivery from Phosphate-Based Glasses. Adv. Drug Delivery Rev..

[cit19] Evans A., Kavanagh K. A. (2021). Evaluation of Metal-Based Antimicrobial Compounds for the Treatment of Bacterial Pathogens. J. Med. Microbiol..

[cit20] El-Fiqi A., Allam R., Kim H.-W. (2021). Antioxidant Cerium Ions-Containing Mesoporous Bioactive Glass Ultrasmall Nanoparticles: Structural, Physico-Chemical, Catalase-Mimic and Biological Properties. Colloids Surf., B.

[cit21] Todorov L., Kostova I., Traykova M. (2019). Lanthanum, Gallium and Their Impact on Oxidative Stress. Curr. Med. Chem..

[cit22] Moghaddam Z., Nery E. T., Unalan I., Hoxha A., Felipe-Sotelo M., Zhao H., Pinna A., Abrahams I., Smales G. J., Boccaccini A. R., Carta D. (2025). Electrospun Porous Phosphate-Based Glass Fibres Containing Gallium and Clove Oil: Cytotoxicity and Antioxidant Properties. Ceram. Int..

[cit23] Abou Néel E. A. , Salih V. and Knowles J. C., Phosphate-Based Glasses, In Comprehensive Biomaterials, Elsevier, 2011, pp. 285–297. 10.1016/B978-0-08-055294-1.00249-X

[cit24] Vallet-Regi M. and Salinas A. J., Mesoporous Bioactive Glasses for Regenerative Medicine, Materials Today Bio, Elsevier B.V., 2021. 10.1016/j.mtbio.2021.100121 PMC8327654 34377972

[cit25] Carta D., Knowles J. C., Smith M. E., Newport R. J. (2007). Synthesis and Structural Characterization of P2O5-CaO-Na2O Sol-Gel Materials. J. Non-Cryst. Solids.

[cit26] Owens G. J. , Singh R. K., Foroutan F., Alqaysi M., Han C. M., Mahapatra C., Kim H. W. and Knowles J. C., Sol-Gel Based Materials for Biomedical Applications, Progress in Materials Science, Elsevier Ltd, 2016, pp. 1–79. 10.1016/j.pmatsci.2015.12.001

[cit27] Hu G. F. (1998). Copper Stimulates Proliferation of Human Endothelial Cells under Culture. J. Cell Biochem..

[cit28] Harris E. D. (2004). Special Article A Requirement for Copper in. Angiogenesis.

[cit29] Li J. , Zhang Y. P. and Kirsner R. S., Angiogenesis in Wound Repair: Angiogenic Growth Factors and the Extracellular Matrix, Microscopy Research and Technique, Wiley-Liss Inc, 2003, pp. 107–114. 10.1002/jemt.10249 12500267

[cit30] Thurman R. B., Gerba C. P., Bitton G. (1989). The Molecular Mechanisms of Copper and Silver Ion Disinfection of Bacteria and Viruses. Crit. Rev. Environ. Control.

[cit31] Kumar M., Mogha N. K., Kumar G., Hussain F., Masram D. T. (2019). Biological Evaluation of Copper(II) Complex with Nalidixic Acid and 2,2′-Bipyridine (Bpy). Inorg. Chim. Acta.

[cit32] Kurtuldu F., Mutlu N., Boccaccini A. R., Galusek D. (2022). Gallium Containing Bioactive Materials: A Review of Anticancer, Antibacterial, and Osteogenic Properties. Bioact. Mater..

[cit33] Pourshahrestani S., Zeimaran E., Adib Kadri N., Gargiulo N., Samuel S., Naveen S. V., Kamarul T., Towler M. R. (2016). Gallium-Containing Mesoporous Bioactive Glass with Potent Hemostatic Activity and Antibacterial Efficacy. J. Mater. Chem. B.

[cit34] Li F., Liu F., Huang K., Yang S. (2022). Advancement of Gallium and Gallium-Based Compounds as Antimicrobial Agents. Front. Bioeng. Biotechnol..

[cit35] Nabavi M., Doeuff S., Sanchez C., Livage J. (1990). Chemical modification of metal alkoxides by solvents: a way to control sol-gel chemistry. J. Non-Cryst. Solids.

[cit36] Kessler V. G., Seisenbaeva G. A. (2023). Molecular Mechanisms of the Metal Oxide Sol-Gel Process and Their Application in Approaches to Thermodynamically Challenging Complex Oxide Materials. J. Sol-Gel Sci. Technol..

[cit37] Deshmukh K., Kovářík T., Křenek T., Docheva D., Stich T., Pola J. (2020). Recent Advances and Future Perspectives of Sol-Gel Derived Porous Bioactive Glasses: A Review. RSC Adv..

[cit38] Lepry W. C. and Nazhat S. N., A Review of Phosphate and Borate Sol–Gel Glasses for Biomedical Applications, Advanced NanoBiomed Research, John Wiley and Sons Inc, 2021. 10.1002/anbr.202000055

[cit39] Smales G. J., Pauw B. R. (2021). The MOUSE Project: A Meticulous Approach for Obtaining Traceable, Wide-Range X-Ray Scattering Information. J. Instrum..

[cit40] Filik J., Ashton A. W., Chang P. C. Y., Chater P. A., Day S. J., Drakopoulos M., Gerring M. W., Hart M. L., Magdysyuk O. V., Michalik S., Smith A., Tang C. C., Terrill N. J., Wharmby M. T., Wilhelm H. (2017). Processing Two-Dimensional X-Ray Diffraction and Small-Angle Scattering Data in DAWN 2. J. Appl. Crystallogr..

[cit41] Pauw B. R., Smith A. J., Snow T., Terrill N. J., Thünemann A. F. (2017). The Modular Small-Angle X-Ray Scattering Data Correction Sequence. J. Appl. Crystallogr..

[cit42] Bressler I., Pauw B. R., Thünemann A. F. (2015). McSAS: Software for the Retrieval of Model Parameter Distributions from Scattering Patterns. J. Appl. Crystallogr..

[cit43] SAXS data for PPG-U deposited on Zenodo 10.5281/zenodo.13709898

[cit44] Brunauer S., Emmett P. H., Teller E. (1938). Adsorption of Gases in Multimolecular Layers. J. Am. Chem. Soc..

[cit45] Brauer D. S. , In Dissolution Behaviour of Phosphate Glasses, Phosphate and Borate Bioactive Glasses, The Royal Society of Chemistry, 2022, pp. 25–43. 10.1039/9781839164750-00025

[cit46] Ahmed I., Lewis M., Olsen I., Knowles J. C. (2004). Phosphate Glasses for Tissue Engineering: Part 1. Processing and Characterisation of a Ternary-Based P2O5–CaO–Na2O Glass System. Biomaterials.

[cit47] Wilkinson H. N., Kidd A. S., Roberts E. R., Hardman M. J. (2021). Human Ex Vivo Wound Model and Whole-Mount Staining Approach to Accurately Evaluate Skin Repair. J. Visualized Exp..

[cit48] Uo M. ; Mizuno M.; Kuboki Y.; Makishima A. and Watari F., Properties and Cytotoxicity of Water Soluble Na O-CaO-P O Glasses, 1998, Vol. 19 10.1016/s0142-9612(98)00136-7 9884040

[cit49] Taye M. B. , Biomedical Applications of Ion-Doped Bioactive Glass: A Review, Applied Nanoscience, Springer Science and Business Media Deutschland GmbH, 2022, pp. 3797–3812. 10.1007/s13204-022-02672-7

[cit50] Valappil S. P., Ready D., Néel E. A. A., Pickup D. M., Chrzanowski W., O’Dell L. A., Newport R. J., Smith M. E., Wilson M., Knowles J. C. (2008). Antimicrobial Gallium-Doped Phosphate-Based Glasses. Adv. Funct. Mater..

[cit51] Foroutan F., McGuire J., Gupta P., Nikolaou A., Kyffin B. A., Kelly N. L., Hanna J. V., Gutierrez-Merino J., Knowles J. C., Baek S. Y., Velliou E., Carta D. (2019). Antibacterial Copper-Doped Calcium Phosphate Glasses for Bone Tissue Regeneration. ACS Biomater. Sci. Eng..

[cit52] Lin Y., Xiao W., Bal B. S., Rahaman M. N. (2016). Effect of Copper-Doped Silicate 13–93 Bioactive Glass Scaffolds on the Response of MC3T3-E1 Cells in Vitro and on Bone Regeneration and Angiogenesis in Rat Calvarial Defects in Vivo. Mater. Sci. Eng. C.

[cit53] Kyffin B. A., Di Pasquale R., Pickup D. M., Foroutan F., Abrahams I., Kanwal N., Keeble D. S., Felipe-Sotelo M., Hoxha A., Moghaddam Z., Hinder S. J., Baker M. A., Nery E. T., Carta D. (2024). Atomic Scale Investigation and Cytocompatibility of Copper and Zinc-Loaded Phosphate-Based Glasses Prepared by Coacervation. Materialia.

[cit54] Mufundirwa A., Sakurai Y., Arao M., Matsumoto M., Imai H., Iwamoto H. (2024). Contrast Variation Method Applied to Structural Evaluation of Catalysts by X-Ray Small-Angle Scattering. Sci. Rep..

[cit55] Wu C., Zhou Y., Xu M., Han P., Chen L., Chang J., Xiao Y. (2013). Copper-Containing Mesoporous Bioactive Glass Scaffolds with Multifunctional Properties of Angiogenesis Capacity, Osteostimulation and Antibacterial Activity. Biomaterials.

[cit56] Arnold C. E., Bordin A., Lawhon S. D., Libal M. C., Bernstein L. R., Cohen N. D. (2012). Antimicrobial Activity of Gallium Maltolate against Staphylococcus Aureus and Methicillin-Resistant S. Aureus and Staphylococcus Pseudintermedius: An in Vitro Study. Vet. Microbiol..

[cit57] Salazar-Alemán D. A., Turner R. J. (2025). Escherichia Coli Growing under Antimicrobial Gallium Nitrate Stress Reveals New Processes of Tolerance and Toxicity. Sci. Rep..

[cit58] Azam A., Ahmed A. S., Oves M., Khan M. S., Habib S. S., Memic A. (2012). Antimicrobial Activity of Metal Oxide Nanoparticles against Gram-positive and Gram-negative Bacteria: A Comparative Study. Int. J. Nanomed..

[cit59] Awais M., Aizaz A., Nazneen A., Bhatti Q. U. A., Akhtar M., Wadood A., Atiq Ur Rehman M. (2022). A Review on the Recent Advancements on Therapeutic Effects of Ions in the Physiological Environments. Prosthesis.

[cit60] Kurtuldu F., Mutlu N., Boccaccini A. R., Galusek D. (2022). Gallium Containing Bioactive Materials: A Review of Anticancer, Antibacterial, and Osteogenic Properties. Bioact. Mater..

[cit61] Łapa A., Cresswell M., Campbell I., Jackson P., Goldmann W. H., Detsch R., Boccaccini A. R. (2020). Gallium- and Cerium-Doped Phosphate Glasses with Antibacterial Properties for Medical Applications. Adv. Eng. Mater..

