# Supplementary material for: Hierarchically porous copper and gallium loaded sol–gel phosphate glasses for enhancement of wound closure
Source: J Mater Chem B. 2025 Nov 14;13(48):15662–77. doi: 10.1039/d5tb01945a (PMC12648381; doi:10.1039/d5tb01945a)
Supplement: TB-013-D5TB01945A-s001 [file TB-013-D5TB01945A-s001.pdf]

Supplementary Information (SI) for Journal of Materials Chemistry B.
This journal is © The Royal Society of Chemistry 2025

SUPPLEMENTARY INFORMATION

Hierarchically porous copper and gallium loaded sol-gel phosphate glasses for enhancement of wound closure

Charlotte A. Berry, [a,b] Katre Reinart, [a] Glen J. Smales, [c, d] Holly N. Wilkinson, [e, f] Matthew J. Hardman [e, f] Sofia Marchesini, [b] William Lee, [b] Eveliny Tomás Nery, [a, g] Zarin Moghaddam, [a] Agron Hoxha, [a] Mónica Felipe-Sotelo, [a] Jorge Gutierrez-Merino, [g] Daniela Carta. [a *]

[a] *School of Chemistry and Chemical Engineering, University of Surrey, Guildford, GU2 7XH, United Kingdom.*

[b] *Surface Technology Group, National Physical Laboratory, Teddington, TW11 0LW, United Kingdom.*

[c] *Bundesanstalt für Materialforschung und –prüfung (BAM), Berlin, Germany.*

[d] *Institute for Inorganic Chemistry, Graz University of Technology, Stremayrgasse 9, 8010 Graz, Austria.*

[e] *Centre for Biomedicine, Hull York Medical School, University of Hull, Hull, HU6 7RX, United Kingdom.*

[f] *Skin Research Centre, Hull York Medical School, University of York, York, YO10 5DD, United Kingdom.*

[g] *School of Biosciences and Medicine, University of Surrey, Guildford, GU2 7XH, United Kingdom.*

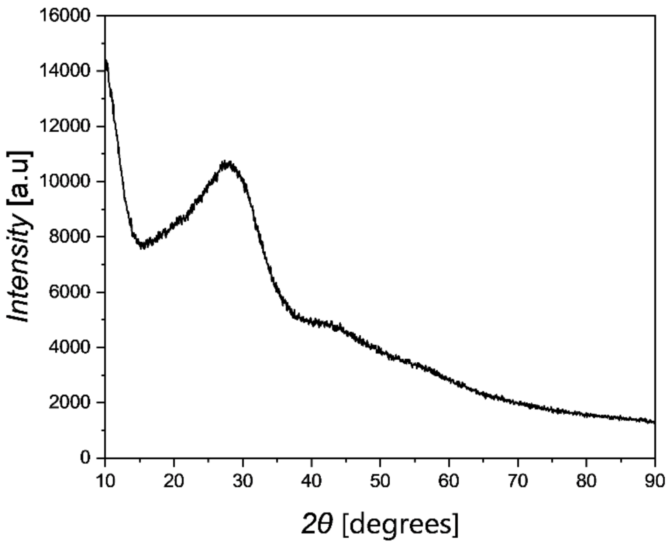

Figure S1. XRD pattern of PPG-U.

Table S1. Nominal composition of PPG-U.

Sample	Composition (mol %)		
	P_2O_5	CaO	Na_2O
PPG-U	48	38	14

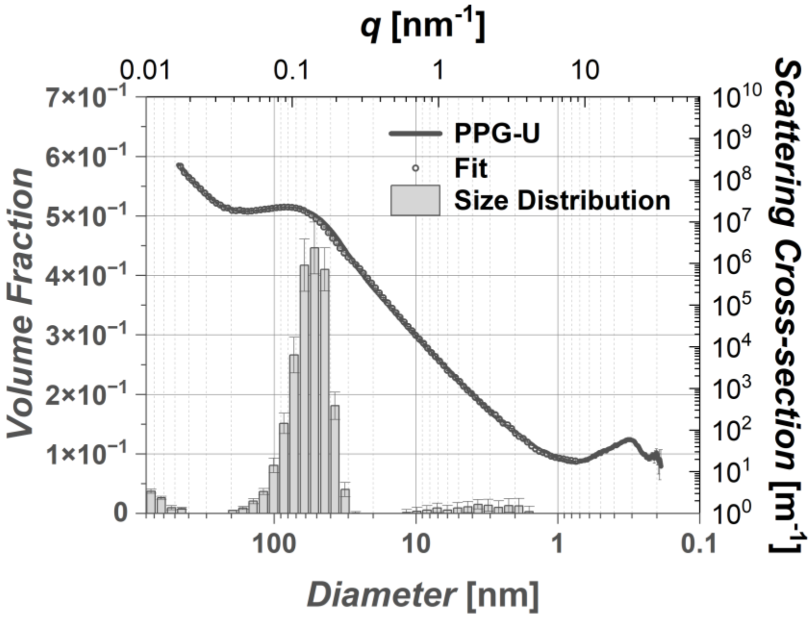

Figure S2. Fitted SAXS data for PPG-U.

Table S2. Mean radii of pore populations from PPG-U derived from fits of SAXS data using Monte-Carlo methods.

Sample	Size distribution range (nm)	
PPG-U	4.2 ± 0.4	60.1 ± 0.2

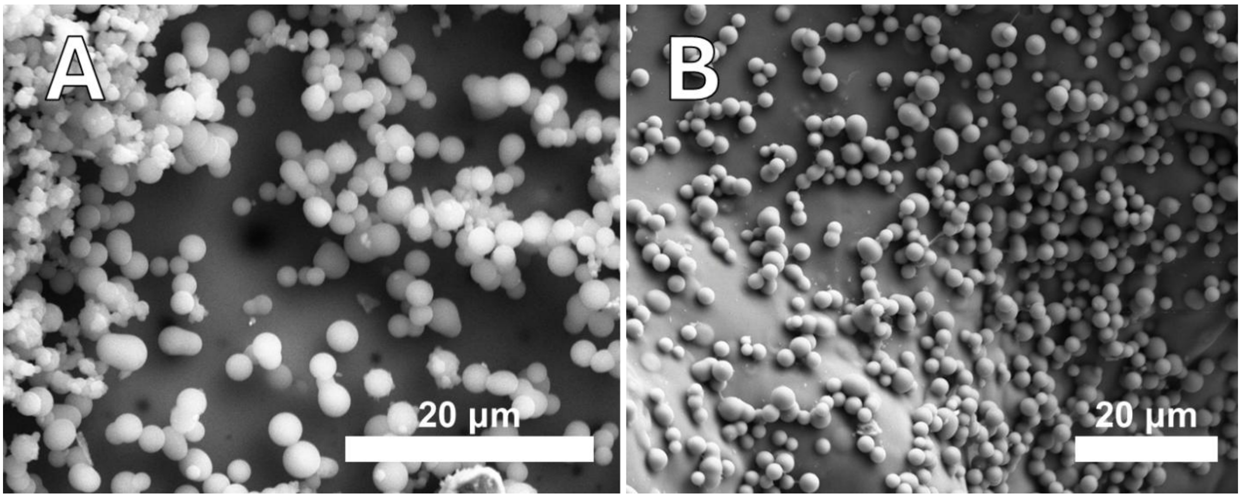

Figure S3. SEM image of A) PPG-U and B) PPG-Ga5 microspheres.

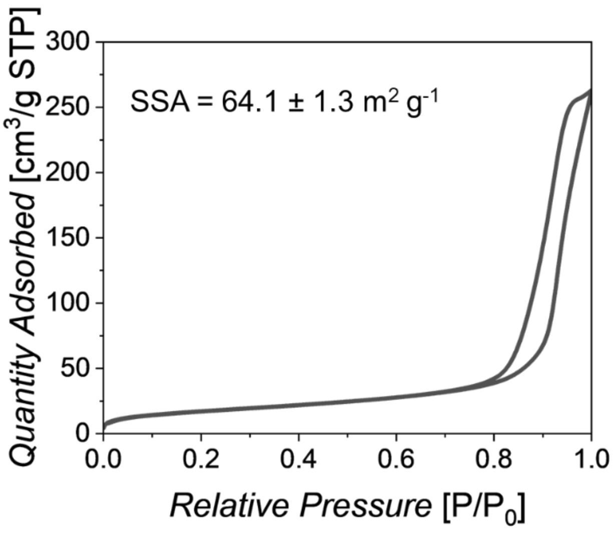

Figure S4. N_2 sorption isotherm of PPG-U.

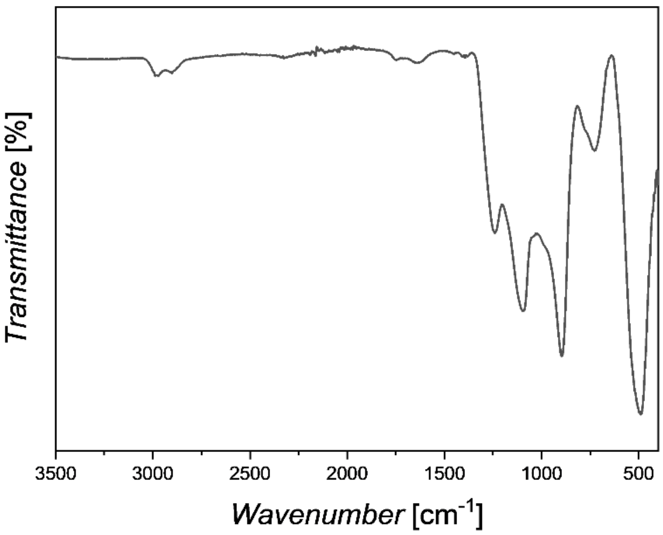

Figure S5. FT-IR spectra of PPG-U.
